# Development of a Machine Learning Model Using Electronic Health Record Data to Identify Antibiotic Use Among Hospitalized Patients

**DOI:** 10.1001/jamanetworkopen.2021.3460

**Published:** 2021-03-29

**Authors:** Rebekah W. Moehring, Matthew Phelan, Eric Lofgren, Alicia Nelson, Elizabeth Dodds Ashley, Deverick J. Anderson, Benjamin A. Goldstein

**Affiliations:** 1 Duke Center for Antimicrobial Stewardship and Infection Prevention, Duke University Medical Center, Durham, North Carolina; 2 Duke Clinical Research Institute, Durham, North Carolina; 3 Department of Biostatistics and Bioinformatics, Duke University, Durham, North Carolina; 4 Paul G. Allen School for Global Animal Health, Washington State University, Pullman

## Abstract

**Importance:**

Comparisons of antimicrobial use among hospitals are difficult to interpret owing to variations in patient case mix. Risk-adjustment strategies incorporating larger numbers of variables haves been proposed as a method to improve comparisons for antimicrobial stewardship assessments.

**Objective:**

To evaluate whether variables of varying complexity and feasibility of measurement, derived retrospectively from the electronic health records, accurately identify inpatient antimicrobial use.

**Design, Setting, and Participants:**

Retrospective cohort study, using a 2-stage random forests machine learning modeling analysis of electronic health record data. Data were split into training and testing sets to measure model performance using area under the curve and absolute error. All adult and pediatric inpatient encounters from October 1, 2015, to September 30, 2017, at 2 community hospitals and 1 academic medical center in the Duke University Health System were analyzed. A total of 204 candidate variables were categorized into 4 tiers based on feasibility of measurement from the electronic health records.

**Main Outcomes and Measures:**

Antimicrobial exposure was measured at the encounter level in 2 ways: binary (ever or never) and number of days of therapy. Analyses were stratified by age (pediatric or adult), unit type, and antibiotic group.

**Results:**

The data set included 170 294 encounters and 204 candidate variables from 3 hospitals during the 3-year study period. Antimicrobial exposure occurred in 80 190 encounters (47%); 64 998 (38%) received 1 to 6 days of therapy, and 15 192 (9%) received 7 or more days of therapy. Two-stage models identified antimicrobial use with high fidelity (mean area under the curve, 0.85; mean absolute error, 1.0 days of therapy). Addition of more complex variables increased accuracy, with largest improvements occurring with inclusion of diagnosis information. Accuracy varied based on location and antibiotic group. Models underestimated the number of days of therapy of encounters with long lengths of stay.

**Conclusions and Relevance:**

Models using variables derived from electronic health records identified antimicrobial exposure accurately. Future risk-adjustment strategies incorporating encounter-level information may make comparisons of antimicrobial use more meaningful for hospital antimicrobial stewardship assessments.

## Key Points

**Question:**

Do variable sets of varying complexity derived from the electronic health record accurately identify inpatient antimicrobial exposure?

**Findings:**

Machine learning models developed in this cohort study identified encounter-level antimicrobial exposures with high fidelity, with a mean area under the curve of 0.85.

**Meaning:**

Encounter-level information from the electronic health record accurately identified antibiotic exposures and may be useful in future risk-adjusted comparisons for hospital antimicrobial stewardship assessments.

## Teaser

This cohort study evaluates whether variables of varying complexity and feasibility of measurement, derived retrospectively from the electronic health records, accurately identify inpatient antimicrobial exposure

## Introduction

Assessment of antimicrobial use (AU) is an essential activity for hospital antimicrobial stewardship programs to identify areas of need and evaluate the effectiveness of interventions. However, evaluating a single hospital’s AU has limited ability to identify improvement opportunities without an external comparator, or benchmark, to indicate where AU may be higher or lower than expected. External benchmark comparisons can identify areas to investigate further, and then hospitals can use more resource-intensive assessments of the appropriateness of AU.^1^

In 2011, the Centers for Disease Control and Prevention National Healthcare Safety Network launched a revised AU Option to collect national data in standardized measures of the number of days of therapy per 1000 days present attributed to a patient care location.^2,3^ In 2015, the Centers for Disease Control and Prevention introduced the Standardized Antimicrobial Administration Ratio (SAAR), a ratio of observed to predicted days of therapy for a particular antimicrobial agent category and location, compared with the national baseline.^4^ The SAAR was endorsed by the National Quality Forum for public health surveillance and internal improvement efforts.^5^ The SAAR models were updated with 2017 data and 7 variables for risk adjustment collected through the National Healthcare Safety Network annual survey: location (eg, medical ward), facility type, teaching status, hospital bed size, number of beds in the intensive care unit, percentage of beds in the intensive care unit, and mean length of stay.^6^

The characteristics of the patients play a large role in risk of antimicrobial exposure, especially comorbid conditions, diagnosis of infection, or indication for antibiotic prophylaxis, which are not directly accounted for in facility- or unit-level variables. Therefore, clinicians who perceive their hospital or unit population as “different” than those used in the national baseline may not believe that the comparisons identify opportunities for improvement. Furthermore, antimicrobial stewardship program teams with limited resources may spend time investigating high SAARs when AU is most associated with case mix instead of appropriateness. Robust strategies for AU risk adjustment are necessary to help remove variation in AU that may not be modifiable by antimicrobial stewardship, making antimicrobial stewardship program assessments and interventions more targeted and efficient.

Identifying variables for risk adjustment of AU must be done carefully, balancing the burden of data collection and standardization. These decisions should consider multiple questions: (1) How feasible is it to collect and report risk-adjustment variables in electronic systems? (2) How accurately do variables identify patient encounters likely to have antibiotic exposures? (3) Is a variable considered reasonable to use for AU risk adjustment by relevant stakeholders?

As an initial step, we aimed to assess how well encounter-level demographic and clinical characteristics identify AU to determine which types of data should be pursued further in risk-adjustment and AU benchmarking. Importantly, this study was not designed for prospective prediction or forecasting. The specific aims were to determine (1) whether models using variables of varying complexity and feasibility of measurement, derived retrospectively from the electronic health record (EHR), can identify inpatient antimicrobial exposures and quantify their number of days of therapy, and (2) how model accuracy differs as the complexity of variable sets increases.

## Methods

### Source Data

We performed a retrospective cohort study using machine learning modeling analyses to estimate 2 outcomes on the encounter level: any exposure to antimicrobials (ever or never) and the number of days of therapy. Existing clinical data from the EHR (Epic) were extracted from inpatient encounters at 2 community hospitals and 1 academic medical center in the Duke University Health System from October 1, 2015, to September 30, 2017. Analyses included any encounter with at least 1 day of exposure to an inpatient unit, including short-stay patients cared for in inpatient areas. Encounters were included if the admission date was within the study time period. Encounters with admission dates occurring prior to the time period were excluded. Encounter information was extracted for the entire encounter even if the discharge date was after September 30, 2017. The study was reviewed and approved by the Duke University institutional review board. Consent was waived owing to use of existing, limited data sets representing minimal risk. The report follows the Strengthening the Reporting of Observational Studies in Epidemiology (STROBE) reporting guideline for cohort studies.

### Clinical Outcomes and Variables

The AU outcomes were classified as ever or never based on the administration of at least 1 dose of a drug from the 2017 SAAR antimicrobial categories (eTable 1 in the Supplement).^3,6^ The numbers of days of therapy were calculated for each antimicrobial group as the sum of calendar days an antimicrobial agent from the group was administered.^3^ Adult (patients aged ≥18 years) and pediatric (patients aged <18 years) encounters were assessed separately. Pediatric encounters of patients cared for on adult units were included in pediatric models and excluded from adult models, and vice versa.

We qualitatively grouped candidate variables into feasibility tiers (Box). Variables considered easier to capture were placed in tier 1; variables considered more difficult to capture were put in progressively higher tiers. Parameterization of variables is further described in the eAppendix in the Supplement.

Box. **Variables Considered in Modeling Analyses of Antimicrobial Use Tiered on Feasibility of Measurement From the Electronic Health Record**

**Tier 1 (Easy)**

Demographic characteristics
  Age
  Sex
  Race
  Ethnicity
Medicare Severity-Diagnosis Related Group by Major Diagnostic Categories
Contextual (season)
  Winter (December-February)
  Spring (March-May)
  Summer (June-August)
  Fall (September-November)
Location or length of stay
  No. of days in a National Healthcare Safety Network location type
  No. of days present in hospital encounter

**Tier 2**

Comorbidities
  Indicators for presence of comorbid diagnoses (by AHRQ CCS code)
  Charlson Comorbidity Index score
  Elixhauser Comorbidity Index score
Acute Events
  Indicators for acute event (by AHRQ CCS code), including indicators for infection diagnoses, immunosuppressed state, and maternity encounter
Procedures
  Indicators of procedures by type (by AHRQ CCS code for *CPT*)

**Tier 3**

Medications
  No. of days of medication exposure by therapeutic class
  Vasoactive medications
  Ever or never received antibiotic in the perioperative areas)
Allergy
  Indicator for antibiotic allergy

**Tier 4 (Hard)**

Laboratory parameters
  White blood cell count
  Platelet count
  Hemoglobin level
  Erythrocyte sedimentation rate
  C-reactive protein level
  Procalcitonin level
  Blood urea nitrogen and creatinine levels
  Estimated glomerular filtration rate
  Albumin level
  Total bilirubin level
  Urinalysis leukocyte esterase, excluding those with >10 squamous cells
Vital signs
  Blood pressure
  Temperature
  Oxygen saturation
  National Early Warning Scores
  SIRS scores
  No. of days of mechanical ventilation
Culture data
  Ever or never culture events
  No. of culture events for encounter, which included body site (eg, urine or blood), positive vs no growth, and multidrug-resistant organism identified (eg, methicillin-resistant *Staphylococcus aureus* or vancomycin-resistant *Enterococcus* species)

Abbreviations: AHRQ, Agency for Healthcare Research and Quality; CCS, Clinical Classifications Software; *CPT*, *Current Procedural Terminology*; SIRS, Systemic Inflammatory Response Syndrome.

### Statistical Analysis

We built machine learning models to assess the added value of different variable sets, first including tier 1 variables, then tiers 1 and 2, tiers 1 through 3, and so forth. We used the machine learning algorithm random forests, a decision tree–based model in which many trees are grown and aggregated together.^7^ This algorithm is able to model nonlinear interactions without prior specification. We used the internally generated out-of-bag error rate to pick the proper tuning parameters.

Many encounters had no antimicrobial use, creating a zero-inflated distribution of days of therapy. Therefore, we used a 2-stage modeling approach.^8^ The first model identified whether an encounter had any administration of an antimicrobial (ever or never). The second model determined how many days of therapy each encounter received in the subset of encounters of patients identified to receive antimicrobials. We set the threshold to move to the days of therapy model at more than 50% probability. This approach produced a probability of any antimicrobial therapy and the estimated days of therapy per encounter. Encounters were separated into an 80% training and 20% testing sets randomly sampled by week of admission date to evenly distribute across time. Model performance was assessed with area under the curve (AUC) based on the ever or never outcome among observations in the testing data set. A higher AUC indicated a stronger performance, or that the model was better able to identify which encounters had patients who received an antimicrobial. The days of therapy model was assessed using absolute error, which indicated the mean number of days of therapy that the model was off for encounters in the testing data set. The closer the absolute error was to zero, the better the accuracy of the models. A total of 360 models were trained (eTable 2 in the Supplement).

## Results

The analysis included 170 294 encounters and 204 variables among 2 community hospitals and 1 academic medical center (Table 1). A total of 80 190 encounters (47%) had antimicrobial exposures; 64 998 (38%) had 1 to 6 days of therapy, and 15 192 (9%) had 7 or more days of therapy (Table 2). Although those with 7 or more days of therapy made up 9% of encounters, these encounters were responsible for 63% of the total number of days of therapy and were more common in the academic medical center. The median number of days of therapy per encounter was 3 (interquartile range, 2-6) but varied by antimicrobial group. A larger number of days of therapy per encounter occurred for antifungals and the hospital-onset agents. Pediatric encounters had a lower prevalence of antimicrobial exposure than did adult encounters (28% vs 50%). Encounters with 0 days of therapy had higher proportions of women, neonatal and childbirth encounters, and short hospital stays. Encounters with 7 or more days of therapy included those with higher rates of comorbidities, long lengths of stay, exposure to medical wards and intensive care units, and Medicare Severity Diagnosis Related Groups (MS-DRG) of respiratory diseases, infectious diseases, and transplant.

**Table 1.** Frequency of Encounters and Days of Therapy by Age Group and Antimicrobial Group

Antimicrobial group^a^	No. (%)		Duration of therapy per encounter with AU, median (IQR), d
	Encounters	Days of therapy	
**Adult encounters**			
Total No.	145 980	417 899	
NHSN-reported agents^b^			
None	72 542 (49.7)	0	0
Any	73 438 (50.3)	417 899 (100)	3 (2-6)
All antibacterials	68 729 (47.1)	383 598 (91.8)	2 (1-4)
Antifungal agents	5148 (3.5)	34 301 (8.2)	4 (2-7)
*C difficile* risk agents	31 241 (21.4)	123 444 (29.5)	3 (2-5)
Community-onset	28 423 (19.5)	99 369 (23.8)	3 (1-4)
Narrow-spectrum beta-lactams	28 141 (19.3)	61 507 (14.7)	2 (1-2)
Hospital-onset	8515 (5.8)	50 985 (12.2)	4 (2-7)
Resistant gram-positive	25 660 (17.6)	91 326 (21.9)	2 (1-4)
**Pediatric encounters**			
Total No.	24 314	65 668	
NHSN-reported agents^b^			
None	17 562 (72.2)	0	0
Any	6752 (27.8)	65 668 (100)	3 (2-7)
All antibacterials	5644 (23.2)	61 335 (93.4)	3 (2-5)
Antifungal agents	431 (1.8)	4333 (6.6)	5 (2-13)
*C difficile* risk agents	2544 (10.5)	15 074 (23)	3 (2-6)
Community-onset			
Broad	1575 (6.5)	5619 (8.6)	2 (1-4)
Narrow	3077 (12.7)	13 554 (20.6)	3 (2-3)
Hospital-onset	1038 (4.3)	11 158 (17)	5 (3-11)
Resistant gram-positive	1615 (6.6)	6937 (10.6)	3 (2-5)
Azithromycin	439 (1.8)	1726 (2.6)	3 (1-5)

Abbreviations: AU, antimicrobial use; *C difficile*, *Clostridioides difficile*; IQR, interquartile range; NHSN, National Healthcare Safety Network; SAAR, standardized antimicrobial administration ratio.

^a^ Based on 2017 SAAR antimicrobial agent categories.

^b^ Indicates any antimicrobial agent reported in the NHSN AU Option. Note that agent group titles and agent lists are included in eTable 1 in the Supplement and that agent categories are not mutually exclusive.

**Table 2.** Encounter Characteristics by Antibacterial Exposure at the Duke Health System

Characteristic	Antimicrobial therapy, No. (%) of encounters (N = 170 294)		
	0 d	1-6 d	≥7 d
No.	90 104	64 998	15 192
Age, y			
<1	11 695 (13)	1950 (3)	653 (4)
1-17	5867 (7)	3172 (5)	977 (6)
18-65	48 274 (54)	34 170 (53)	8201 (54)
>65	24 268 (27)	25 706 (40)	5361 (35)
Female sex	51 212 (57)	34 514 (53)	7165 (47)
Hospital			
Academic medical center	52 723 (59)	37 422 (58)	10 729 (71)
Community hospital 1	25 382 (28)	14 003 (22)	2679 (18)
Community hospital 2	11 999 (13)	13 573 (21)	1784 (12)
Location			
Labor ward	8995 (10)	2177 (3)	178 (1)
Neurology ward	2464 (3)	2449 (4)	385 (3)
Neurosurgery ward	3952 (4)	3856 (6)	611 (4)
Surgery ward	14 924 (17)	17 059 (26)	3545 (23)
Medical ward	40 213 (45)	34 119 (52)	8923 (59)
Medical or surgical critical care	4595 (5)	5775 (9)	3864 (25)
Pulmonary ward	974 (1)	1159 (2)	830 (5)
Hematopoietic stem cell transplant ward	270 (<1)	527 (1)	724 (5)
Length of stay, No. of days present			
1	8811 (10)	2585 (4)	0
2	25 637 (28)	14 446 (22)	18 (<1)
3	22 003 (24)	12 895 (20)	192 (1)
4-7	25 724 (29)	24 381 (38)	3757 (25)
8-14	6212 (7)	7922 (12)	5231 (34)
>15	1717 (2)	2769 (4)	5994 (39)
Charlson Comorbidity Index category			
Cerebrovascular disease	9134 (10)	5861 (9)	2099 (14)
Peptic ulcer disease	2268 (3)	1560 (2)	689 (5)
Hemiplegia or paraplegia	2966 (3)	2156 (3)	1026 (7)
Diabetes without complication	18 877 (21)	16 188 (25)	5003 (33)
Diabetes with complication	10 434 (12)	8634 (13)	3111 (20)
Metastatic tumor	5552 (6)	5057 (8)	1281 (8)
Malignancy	5800 (6)	4743 (7)	1943 (13)
Peripheral vascular disease	9229 (10)	7867 (12)	2739 (18)
Mild liver disease	5800 (6)	4743 (7)	1943 (13)
Moderate or severe liver disease	1548 (2)	1783 (3)	822 (5)
Kidney disease	15 775 (18)	13 822 (21)	4813 (32)
COPD	17 787 (20)	16 704 (26)	4974 (33)
Dementia	3606 (4)	3747 (6)	994 (7)
AIDS	389 (<1)	423 (1)	201 (1)
DRG MDC			
Newborns or neonates	10 400 (12)	1423 (2)	362 (2)
Pregnancy or childbirth	10 382 (12)	2253 (3)	98 (1)
Endocrine, nutritional, or metabolic	3500 (4)	1429 (2)	263 (2)
Nervous system	7368 (8)	3837 (6)	804 (5)
Digestive system	6680 (7)	3533 (5)	1127 (7)
Blood or blood-forming organs	1626 (2)	1005 (2)	459 (3)
Kidney or urinary tract	2242 (2)	4010 (6)	658 (4)
Respiratory system	3363 (4)	6268 (10)	1793 (12)
Skin, subcutaneous tissue, or breast	441 (<1)	1532 (2)	331 (2)
Musculoskeletal system	3251 (4)	13 277 (20)	985 (6)
Infectious or parasitic disease	581 (1)	4139 (6)	2880 (19)
Transplant	116 (<1)	433 (1)	1425 (9)
Missing	13 594 (15)	9796 (15)	2259 (15)

Abbreviations: COPD, chronic obstructive pulmonary disease; DRG, Diagnosis Related Group; MDC, Major Diagnostic Categories.

**Figure 1.**
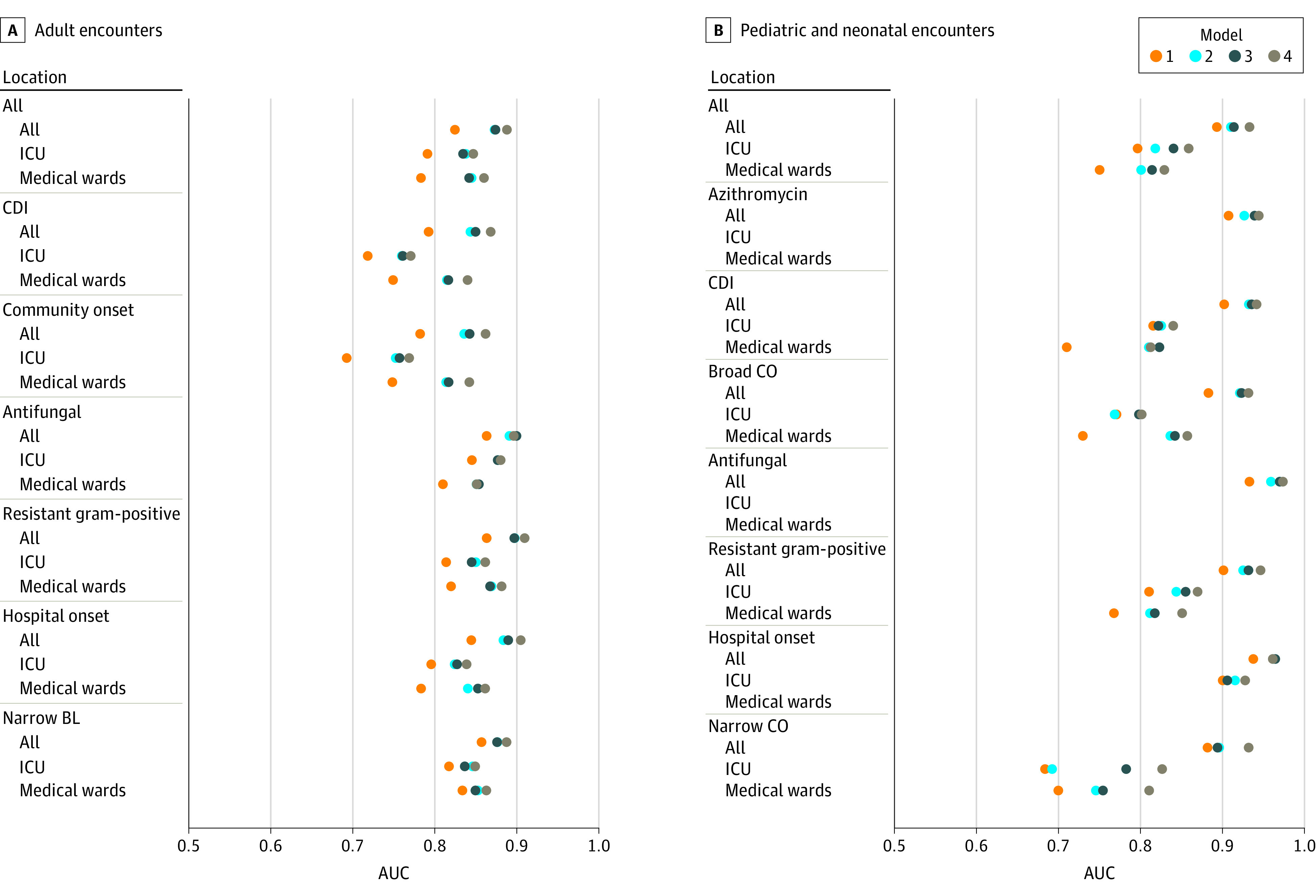
Model Performance When Identifying Ever Receiving an Antimicrobial During the Encounter by Age, Location, Antimicrobial Group, and Input Variable Feasibility Tier A, Model output for adult encounters. B, Model output for pediatric and neonatal encounters. Each data point represents a unique model built based on location, feasibility tier of variables used, antimicrobial group, and adult or pediatric populations. The closer the AUC value is to 1, the better the model was at classifying whether antimicrobials were administered. Some location strata in the analysis of pediatric encounters were too small to fit a model. In these scenarios, only estimates for the “all locations” category were reported. Antimicrobial groups and agents are listed in eTable 1 in the Supplement. AUC indicates area under the curve; BL, beta-lactam; CDI, *Clostridioides difficile* infection risk agents; CO, community onset; and ICU, intensive care unit.

**Figure 2.**
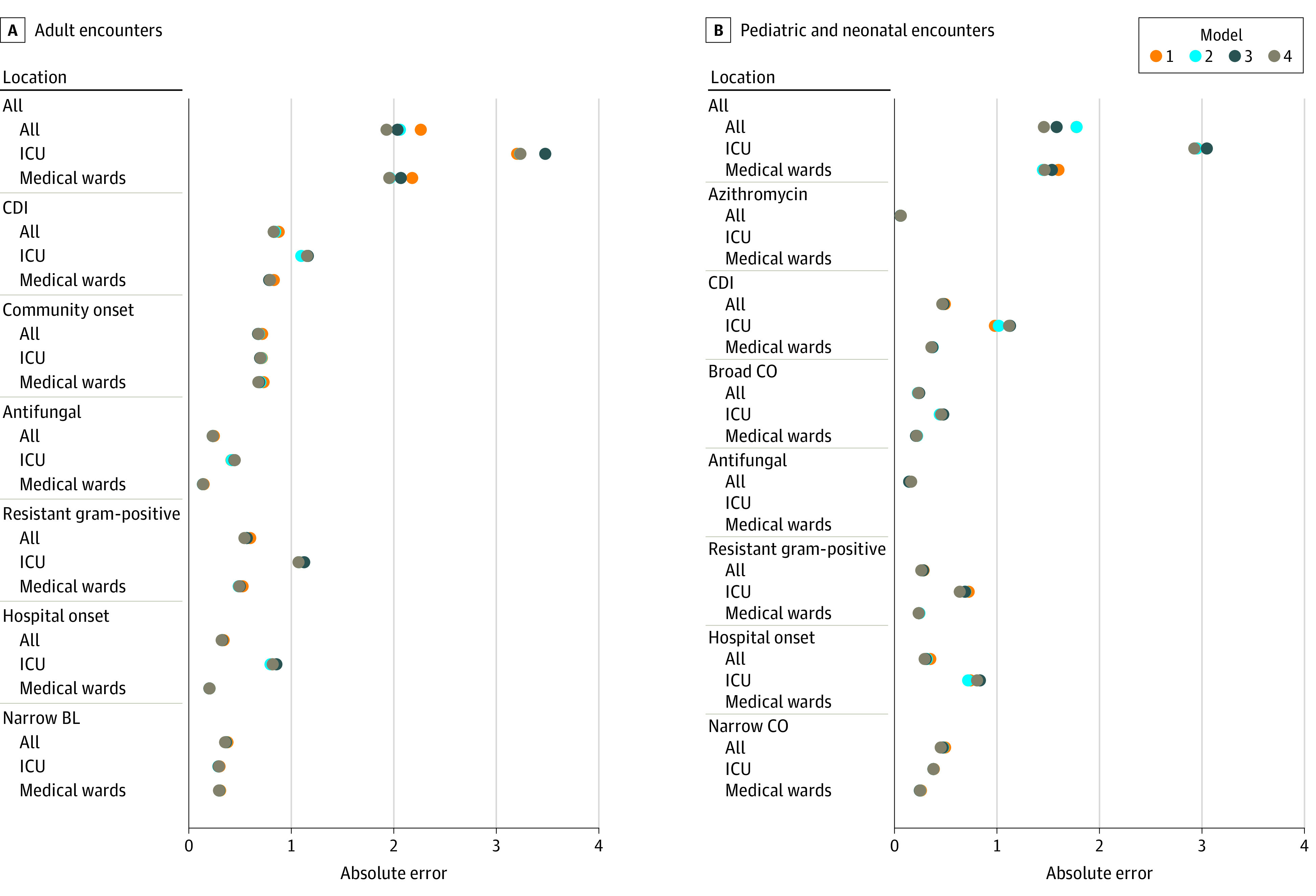
Model Performance When Identifying Days of Therapy of Antimicrobials During the Encounter by Age, Location, Antimicrobial Group, and Input Variable Feasibility Tier A, Model output for adult encounters. B, Model output for pediatric and neonatal encounters. Each data point represents a unique model built based on location, feasibility tier of variables used, antimicrobial group, and adult or pediatric populations. The closer the mean absolute error is to 0, the better the model was at estimating the number of days of antimicrobial therapy. Some location strata in the analysis of pediatric encounters were too small to fit a model. In these scenarios, only estimates for the “all locations” category were reported. Antimicrobial groups and agents are listed in eTable 1 in the Supplement. BL indicates beta-lactam; CDI, *Clostridioides difficile* infection risk agents; CO, community onset; and ICU, intensive care unit.

Models accurately identified antimicrobial exposure in the testing data set; the majority of AUCs were above 0.8, with a mean AUC of 0.85 (Figure 1; eTable 4 and eTable 5 in the Supplement). Mean AUCs were similar for adult and pediatric models and antimicrobial groups; however, adult models showed less variation (AUC SDs of 0.04 vs 0.07), likely owing to larger sample size. The simplest input variables (tier 1) yielded the lowest AUCs. As complexity of the input variables increased, AUCs improved. In some SAAR groups, AUC improved by more than 0.1 when using more complex variable sets. The largest improvements in accuracy were found when more granular information on diagnoses were included in the variable sets (eg, moving from tier 1, which used 20 MS-DRG Major Diagnostic Categories, to tier 2, which used 225 Agency for Healthcare Research and Quality Clinical Classifications Software categories). The days of therapy models had mean absolute errors of approximately 1 day of therapy, whereas the all antibacterial category had errors between 2 and 3.5 days of therapy (Figure 2). Additional accuracy associated with more complex variable sets was not as evident in the days of therapy absolute errors as in ever or never AUCs.

We observed variability in model performance based on antimicrobial group and location, which seemed to be associated with frequency of use. The days of therapy model performance was worse in the all antibacterial, intensive care unit–specific models in which AU was more frequent and population smaller. This same pattern was not necessarily found when looking at other groups. For example, the resistant Gram-positive group had slightly worse performance than the narrow beta-lactam group in adults, even though prevalence of use was similar (17% and 19%, respectively).

Models were less accurate in estimating the numbers of days of therapy for encounters with extremely high numbers of days of therapy, which may occur in encounters with extremely long lengths of stay or those involving an organ transplant. We evaluated the all antibacterials, tier 4 days of therapy model for calibration to encounters with lengths of stay greater than the 90th percentile of 10 days (eFigure in the Supplement). The model underestimated the true number of days of therapy in the higher quantiles of the length of stay distribution, with an overall calibration slope of 0.91, where 1.0 indicates perfect calibration. To better estimate the effect of high outliers, a sensitivity analysis was performed that looked at model performance in several subpopulations with high numbers of days of therapy and/or long lengths of stay. In both subgroups of days of therapy greater than the 90th quantile and length of stay greater than the 90th quantile, absolute errors increased to 11 days of therapy.

## Discussion

Our study used encounter-level variables retrospectively derived from the EHR to identify AU. Models were able to quantify both ever or never exposures and days of therapy with high fidelity (mean AUC, 0.85; mean error, 1.0 days of therapy). Encounters with a high number of days of therapy and a long length of stay were harder to quantify. The largest accuracy improvements were seen when more granularly measured information on diagnosis were included in the variable sets. We did not find, however, that the most complex variables, including nonantibiotic medications (tier 3), laboratory data, and vital signs (tier 4), substantially improved accuracy. Simpler variable sets performed adequately without the addition of the most complex data. Our analyses suggest that additional variables retrospectively captured in the EHR may be used in AU risk-adjustment strategies to improve comparisons among hospitalized populations with differing characteristics.

Prior investigations in AU prediction and risk-adjustment have rarely included information on encounter-level, EHR-derived factors. Multiple prior investigators used facility- or unit-level aggregate data to provide risk-adjusted estimates. Previously explored factors included unit type, clinical service line, hospital teaching status, case-mix index, or summarized measures of patient characteristics (eg, proportion of encounters with patients >65 years of age).^1,4,9,10,11^ Previously, encounter-level modeling of AU was investigated by Yu et al among 35 hospitals in the Kaiser Permanente system.^12^ The Kaiser Permanente investigators presented 3 strategies for producing hospital AU observed to expected ratios: (1) a “complex” encounter-level model using 27 factors, (2) a “simplified” encounter-level model using the 5 most significant factors from the complex model, and (3) a “facility” model using data aggregated to the hospital level and 2 factors from the 2014 SAAR strategy (location and teaching status). Comparing the complex strategy with the simplified strategy did not appreciably change the observed to expected ratios for individual hospitals, but comparing encounter-level models to the facility strategy resulted in larger divergence in observed to expected ratios. The Kaiser Permanente investigators found that the most influential factor in the encounter-level models was DRG, which they grouped in 4 categories based on associations of each DRG with AU in each antibiotic group. More recently, Goodman et al evaluated AU among 576 hospitals in the Premier Database using negative binomial regression and encounter-level variable sets for specified comorbidities based on *International Statistical Classification of Diseases and Related Health Problems, Tenth Revision* (*ICD-10*) codes.^13^ Models using encounter-level characteristics demonstrated 24% higher accuracy compared with those using only facility-level characteristics.

Although it is difficult to directly compare our approach with prior models, our study supports the conclusion that encounter-level information, especially information on diagnosis, provides us with the additional and robust ability to identify antibiotic exposures. Our analysis demonstrated that the use of a larger number of granular diagnosis categories was associated with improved model accuracy: Tier 2 models included 225 Agency for Healthcare Research and Quality Clinical Classifications Software categories compared with 20 MS-DRG Major Diagnostic Categories groups in tier 1.^14^ Thus, evaluation of diagnosis information should be a key part of future development work in AU risk adjustment. In contrast, our findings suggest that incorporating complex variables, including laboratory and nonantibiotic medication data, may not be very fruitful in achieving additional accuracy.

Unlike prior literature, we used a 2-stage machine learning approach instead of regression, which provided several analytic advantages. First, our 2-stage modeling approach allowed us to address 2 separate questions: Was an encounter likely to receive antibiotics? If so, how much? Our results showed differences in our ability to answer these 2 questions, with the former benefitting from a larger variable set that included detailed diagnosis categories. We speculate that the choice of whether to use antibiotics is more standardized and thus easier to model with structured EHR data. However, the specific number of days of therapy was more difficult to model. The number of days of therapy may be more subjective and random, which may explain why adding additional complexity does not seem to improve accuracy. The 2-stage approach directly addressed the heavily zero-inflated nature of encounter-level AU data and further highlighted the effect of high outliers. Second, we used a separate validation data set to provide distinct estimates of model accuracy. Prior reports have instead relied on measures of variance (*R*^2^ or pseudo-*R*^2^ values), comparisons with raw AU estimates, or comparisons of one modeling strategy with another without an estimate of accuracy.^6,9,12,15^ We believe AUC and absolute error estimates of accuracy from this investigation could be used as a starting point to compare against future models for AU risk adjustment on the encounter level. Finally, machine learning algorithms allowed for the inclusion of many potential variables without requirements to prespecify interactions and collinearity, thus avoiding subjective interim modeling decisions that are analyst dependent and likely providing superior accuracy. Importantly, machine learning is not required for risk-adjustment modeling; however, machine learning methods did allow us to evaluate larger numbers of candidate variables compared with prior literature.

The aim of this analysis was to evaluate whether EHR-derived variables in tiered feasibility sets were associated with improved accuracy in identifying AU. Accuracy, however, is not the only factor to consider. Clinicians and other end users may be uncomfortable using risk-adjustment variables that do not have an established causal understanding of how they associate with AU. Factors modified by the quality of antimicrobial stewardship would not be appropriate for risk-adjustment purposes. For example, some diagnoses measured at the end of the hospital stay may occur as a complication of care or as a result of antimicrobial stewardship quality (eg, hospital-onset infections), and thus they should be considered for exclusion from risk adjustment. Diagnosis claims data generated at the end of a hospital stay, however, are routinely used for quality assessments for outcomes such as mortality and readmission due to feasibility advantages of standardized data.^16^ We believe that future research should focus on understanding epidemiologic associations with AU as well as consensus-building to determine which factors, and diagnoses, are considered reasonable for use in AU risk adjustment. This future work is especially important if the SAAR has the potential to become a publicly reported metric.

In our study, encounters with 7 or more days of therapy made up only 9% of hospital encounters but 63% of days of therapy. This finding suggests that high outlier encounters may be very influential in aggregate measures of AU and may explain some of the variability seen in comparisons of AU. Furthermore, our models had the most difficulty identifying encounters with a high number of days of therapy and a long length of stay, with absolute errors of 11 days of therapy among the highest quantiles. Encounters with long lengths of stay occurred most frequently in the academic hospital in our study, likely associated with specific case mix and practice, including transplant recipients. However, high outlier encounters occurring in small hospitals may be even more disruptive to aggregate AU rates. This highlights the need for risk adjustment and improved methods to identify encounter characteristics associated with extremes of antibiotic exposure. Future modeling investigations, as well as antibiotic stewardship strategies, could focus on high outlier subgroups as a targeted population of interest.

### Limitations

Limitations are important to acknowledge. First, the data set and tiered feasibility schema were limited to experiences and population within 2 Duke Health System hospitals and EHR data and may not be generalizable to other practice settings. The findings of this study should be validated in larger and more diverse samples. Second, we used a machine learning approach to address the size and complexity of the input data sets, which did not provide information on direction and degree of effects from individual variables. As already discussed, these associations would be important for clinical stakeholders who use AU and risk-adjusted estimates to make decisions about antibiotic stewardship strategy. Dedicated epidemiologic investigations would be better suited to establish estimates of association and theories of causality. Third, we used previously defined groups of *ICD-10* codes and *Current Procedural Terminology* codes that were not expressly designed for the purpose of AU risk adjustment. Efforts to categorize inpatient codes into levels of “appropriateness” for risk adjustment could be pursued, similar to efforts recently published by Chua et al to categorize outpatient *ICD-10* codes into categories of appropriateness for antibiotic exposure.^17^ Importantly, diagnosis categories that are thought to be associated with the quality of antibiotic stewardship should be better defined. We used outcome definitions and antimicrobial groups from the National Healthcare Safety Network AU Option. However, our aims, unit of analysis, patient population, methods, and measures for model performance were different (eTable 3 in the Supplement).

## Conclusions

More development will be required before applying encounter-level AU risk adjustment for the assessment of stewardship practice. Importantly, inclusion of encounter-level data in national AU benchmarking strategies must overcome feasibility barriers, must carefully consider practical tradeoffs, and should include input from relevant stakeholders to establish which encounter-level factors would be best used in a future risk-adjustment strategy. Our analyses using encounter-level data were able to identify which encounters of patients received antibiotics accurately, and we hope that they can inform those making such judgements about the accuracy tradeoff that may result from excluding complex variables. We believe that including encounter-level data in risk-adjustment models would produce more meaningful comparisons to develop an antimicrobial stewardship program strategy and improve the efficiency of antimicrobial stewardship practice assessments.

## Notes

Supplement. **eTable 1.** Adult and Pediatric Antimicrobial Groups **eAppendix.** Parameterization of Variables **eTable 2.** Summary of Models Created to Identify Antibiotic Exposure on the Encounter Level **eFigure.** Model Calibration for Encounters With Lengths of Stay Greater Than 10 Days **eTable 3.** Compare and Contrast Current Analyses and 2017 SAAR Method **eTable 4.** Adult Models AUC Values **eTable 5.** Pediatric Models AUC Values **eReferences.**
