# Supplementary material for: Development of a Machine Learning Model Using Electronic Health Record Data to Identify Antibiotic Use Among Hospitalized Patients
Source: JAMA Netw Open. 2021 Mar 29;4(3):e213460. doi: 10.1001/jamanetworkopen.2021.3460 (PMC8008288; doi:10.1001/jamanetworkopen.2021.3460)
Supplement: Supplement. — eTable 1. Adult and Pediatric Antimicrobial Groups eAppendix. Parameterization of Variables eTable 2. Summary of Models Created to Identify Antibiotic Exposure on the Encounter Level eFigure. Model Calibration for Encounters With Lengths of Stay Greater Than 10 Days eTable 3. Compare and Contrast Current Analyses and 2017 SAAR Method eTable 4. Adult Models AUC Values eTable 5. Pediatric Models AUC Values eReferences. [file jamanetwopen-e213460-s001.pdf]

Supplemental Online Content

Moehring RW, Phelan M, Lofgren E, et al. Development of a machine learning model using electronic health record data to identify antibiotic use among hospitalized patients. *JAMA Netw Open*. 2012;4(3):e213460. doi:10.1001/jamanetworkopen.2021.3460

eTable 1. Adult and Pediatric Antimicrobial Groups

eAppendix. Parameterization of Variables

eTable 2. Summary of Models Created to Identify Antibiotic Exposure on the Encounter Level

eFigure. Model Calibration for Encounters With Lengths of Stay Greater Than 10 Days

eTable 3. Compare and Contrast Current Analyses and 2017 SAAR Method

eTable 4. Adult Models AUC Values

eTable 5. Pediatric Models AUC Values

eReferences.

This supplemental material has been provided by the authors to give readers additional information about their work.

© 2021 Moehring RW et al. *JAMA Network Open.*

eTable 1. Adult and Pediatric Antimicrobial Groups

Adult or Pediatric	Antimicrobial Group Name Used in Tables and Figures (Abbreviation)	NHSN Antimicrobial Agent Category Title	Included Agents
Adult	All antibacterials	Adult All antibacterial agents	
	Anti-fungal	Adult Antifungal agents predominantly used for invasive candidiasis	FLUCONAZOLE<br>ANIDULAFUNGIN<br>CASPOFUNGIN<br>MICAFUNGIN
	C. difficile agents (CDI)	Adult Antibacterial agents posing the highest risk for CDI	CLINDAMYCIN<br>CIPROFLOXACIN<br>GEMIFLOXACIN<br>LEVOFLOXACIN<br>MOXIFLOXACIN<br>CEFOTAXIME<br>CEFTRIAXONE<br>CEFTAZIDIME<br>CEFEPIME<br>CEFDINIR<br>CEFPODOXIME<br>CEFIXIME
	Community-onset	Adult Broad spectrum antibacterial agents predominantly used for community-acquired infections	CEFOTAXIME<br>CEFTRIAXONE<br>CIPROFLOXACIN<br>ERTAPENEM<br>GEMIFLOXACIN<br>LEVOFLOXACIN<br>MOXIFLOXACIN<br>CEFDINIR<br>CEFPODOXIME<br>CEFIXIME<br>CEFUROXIME<br>CEFACLOR<br>CEFPROZIL
	Hospital-onset	Adult Broad spectrum antibacterial agents predominantly used for hospital-onset infections	GENTAMICIN (IV only)<br>TOBRAMYCIN (IV only)<br>AMIKACIN (IV only)<br>PIPERACILLIN/TAZOBACTAM<br>CEFTAZIDIME<br>CEFEPIME<br>IMIPENEM/CILASTATIN<br>MEROPENEM<br>AZTREONAM (IV)<br>DORIPENEM
	Narrow spectrum beta-lactam (Narrow BL)	Adult Narrow spectrum beta-lactam agents	CEFAZOLIN<br>CEFOTETAN<br>CEFOXITIN<br>AMPICILLIN<br>AMOXICILLIN<br>AMOXICILLIN/CLAVULANATE<br>AMPICILLIN/SULBACTAM

© 2021 Moehring RW et al. *JAMA Network Open.*

Adult or Pediatric	Antimicrobial Group Name Used in Tables and Figures (Abbreviation)	NHSN Antimicrobial Agent Category Title	Included Agents
			CEPHALEXIN<br>CEFADROXIL<br>PENICILLIN G<br>PENICILLIN V<br>DICLOXACILLIN<br>NAFCILLIN<br>OXACILLIN
	Resistant gram positive	Adult Antibacterial agents predominantly used for resistant Gram-positive infections (e.g., MRSA)	CEFTAROLINE<br>DALBAVANCIN<br>DAPTOMYCIN<br>LINEZOLID<br>ORITAVANCIN<br>QUINUPRISTIN/DALFOPRISTIN<br>TEDIZOLID<br>TELAVANCIN<br>VANCOMYCIN (IV only)
Pediatric	All Antibacterials	Pediatric All antibacterial agents	
	Anti-fungal	Pediatric Antifungal agents predominantly used for invasive candidiasis	FLUCONAZOLE<br>ANIDULAFUNGIN<br>CASPOFUNGIN<br>MICAFUNGIN
	Azithromycin	Pediatric Azithromycin	AZITHROMYCIN
	C. difficile agents (CDI)	Pediatric Antibacterial agents posing the highest risk for CDI	CLINDAMYCIN<br>CIPROFLOXACIN<br>GEMIFLOXACIN<br>LEVOFLOXACIN<br>MOXIFLOXACIN<br>CEFOTAXIME<br>CEFTRIAXONE<br>CEFTAZIDIME<br>CEFEPIME<br>CEFDINIR<br>CEFPODOXIME<br>CEFIXIME
	Broad Community-onset (Broad CO)	Pediatric Broad spectrum antibacterial agents predominantly used for community-acquired infections	CEFOTAXIME<br>CEFTRIAXONE<br>AMPICILLIN/SULBACTAM<br>AMOXICILLIN/CLAVULANATE<br>CEFDINIR<br>CEFIXIME<br>CEFPODOXIME<br>CEFUROXIME<br>CEFACLOR<br>CEFPROZIL
	Narrow Community-onset (Narrow CO)	Pediatric Narrow spectrum beta-lactam agents Pediatric Azithromycin	AMPICILLIN<br>AMOXICILLIN<br>PENICILLIN G<br>PENICILLIN V

© 2021 Moehring RW et al. *JAMA Network Open.*

Adult or Pediatric	Antimicrobial Group Name Used in Tables and Figures (Abbreviation)	NHSN Antimicrobial Agent Category Title	Included Agents
			CEPHALEXIN<br>CEFAZOLIN<br>CEFADROXIL<br>NAFCILLIN<br>OXACILLIN<br>DICLOXACILLIN<br>CEFOXITIN<br>CEFOTETAN
	Hospital-onset	Pediatric Broad spectrum antibacterial agents predominantly used for hospital-onset infections	AMIKACIN (IV only)<br>TOBRAMYCIN (IV only)<br>AZTREONAM (IV only)<br>CEFEPIME<br>CEFTAZIDIME<br>PIPERACILLIN/TAZOBACTAM<br>ERTAPENEM<br>DORIPENEM<br>IMIPENEM/CILASTATIN<br>MEROPENEM<br>CIPROFLOXACIN<br>LEVOFLOXACIN<br>MOXIFLOXACIN<br>GEMIFLOXACIN
	Gram positive	Pediatric Antibacterial agents predominantly used for resistant Gram-positive infections (e.g., MRSA)	CEFTAROLINE<br>DAPTOMYCIN<br>LINEZOLID<br>VANCOMYCIN (IV only)<br>CLINDAMYCIN<br>DALBAVANCIN<br>ORITAVANCIN<br>QUINUPRISTIN/DALFOPRISTIN<br>TELAVANCIN<br>TEDIZOLID

IV=intravenous route

© 2021 Moehring RW et al. *JAMA Network Open.*

eAppendix. Parameterization of Variables

Location by NHSN category has been previously employed in risk-adjustment.[1] We defined each encounter by the cumulative number of days in each NHSN location. For example, if a patient spent 3 days in the medical ICU then 5 days on the medical ward, their encounter would assign 3 days attributed to the medical ICU and 5 days attributed to medical ward; the day of transfer between units would be counted in both unit-specific counts. A measure for total hospital length of stay, or days present in any inpatient unit, was also included. Analyses stratified by location were limited to resemble the 2014 SAAR location types including encounters with 1) days spent on a medical, medical/surgical, or surgical critical care unit, 2) days spent on a medical, medical/surgical, or surgical ward, and 3) days spent in any NHSN inpatient location.[1,2]

Comorbidities and events occurring during the encounter were defined by encounter diagnoses and procedure codes. International Classifications of Diseases 10 (ICD-10) diagnoses were grouped by the Agency for Healthcare Research and Quality Clinical Classification Software (AHRQ CCS) groups and in Charlson, Elixhauser, and Medicare Severity-Diagnosis Related Groups (MS-DRG).[3,4] MS-DRGs were further grouped by Centers for Medicare and Medicaid Major Diagnosis Category (MDC).[3] We derived a four-category seasonality variable by month of admission date. Variables in time-varying format were summarized by encounter. Vital sign measurements were expressed as minimum, median, mean, maximum over the encounter. Lab results were summarized as the number of times measured, number of high days, number of low days, number of normal days, and number of unknown days. For National Early Warning Score (NEWS) and Systemic Inflammatory Response Syndrome (SIRS) scores, we used days greater than threshold value of 7 and 2, respectively. Non-antimicrobial medications were grouped by therapeutic class defined in the EHR and measured as an ever/never exposure and the number days administered.

© 2021 Moehring RW et al. *JAMA Network Open.*

eTable 2. Summary of Models Created to Identify Antibiotic Exposure on the Encounter Level

Age group + Antimicrobial group (15)	Location (3)	Feasibility Tier (4)	Outcomes (2)
Adult + All antibacterials<br>Adult + Anti-fungal<br>Adult + *C. difficile* agents<br>Adult + Community-onset<br>Adult + Hospital-onset<br>Adult + Narrow spectrum beta lactam<br>Adult + Resistant Gram-positive<br>Pediatric + All antibacterials<br>Pediatric + Anti-fungal<br>Pediatric + Azithromycin<br>Pediatric + *C. difficile* agents<br>Pediatric + Broad Community-onset<br>Pediatric + Narrow Community-onset<br>Pediatric + Hospital-onset<br>Pediatric + Resistant Gram-positive	Any<br>ICU<br>Ward	1-Easy<br>2<br>3<br>4-Hard	Ever/Never<br>Days of therapy

Note the corresponding NHSN Antimicrobial category titles and agent lists are included in eTable 1.

Any location includes all inpatient areas mapped to an NHSN inpatient unit category. ICU locations included NHSN inpatient unit category specified as medical, medical/surgical, or surgical critical care. Ward locations included NHSN inpatient unit category specified as medical, surgical, or medical/surgical ward. This approach is different than the 2017 NHSN SAAR method (eTable 3).

© 2021 Moehring RW et al. *JAMA Network Open.*

eFigure. Model Calibration for Encounters With Lengths of Stay Greater Than 10 Days.

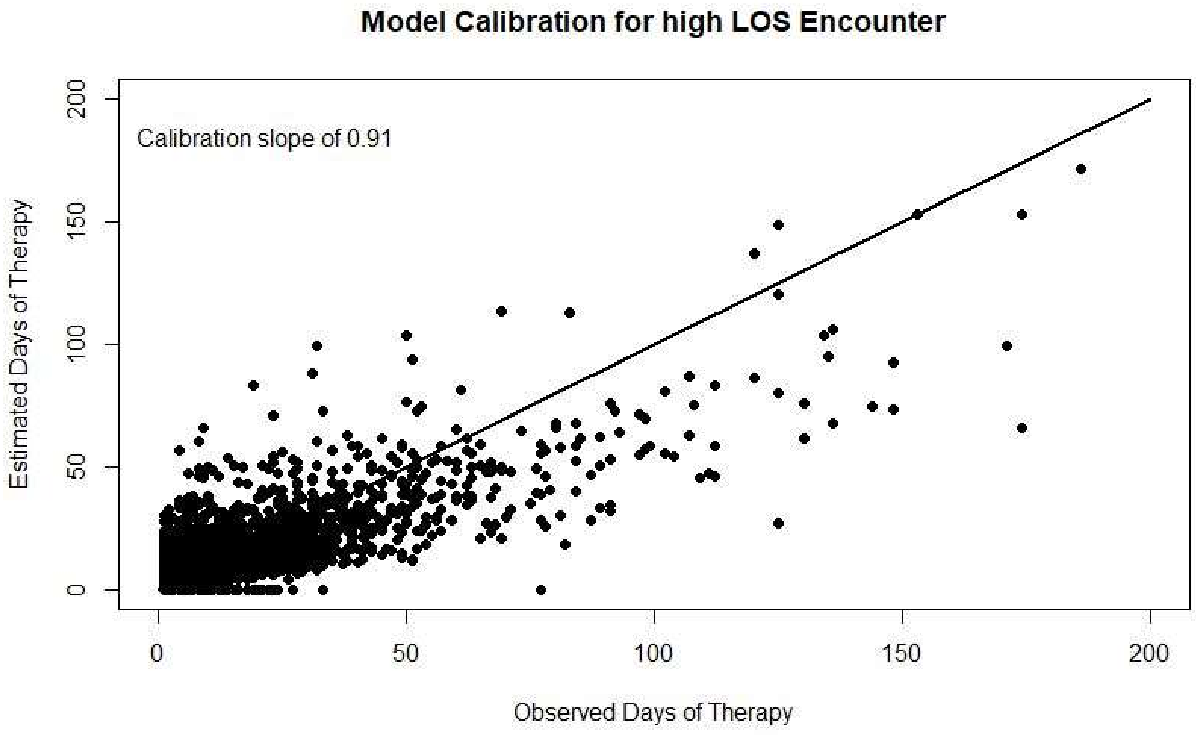

A calibration slope of 1.0 indicates perfect accuracy. The model used to produce the estimated days of therapy was the all antibacterial, adult, all locations model. LOS=length of stay

© 2021 Moehring RW et al. *JAMA Network Open.*

eTable 3. Compare and Contrast Current Analyses and 2017 SAAR Method

Compare/Contrast	2017 SAAR Method	Current Analyses
Same	Antimicrobial agent categories (see eTable 1).<br>Adult and pediatric modeled separately.<br>Outcome in days of therapy (DOT).	
Different: Unit of analysis	Location-time (in month, quarter, or year)	Inpatient encounter
Different: Modeling strategy	Negative binomial models estimating days of therapy with offset of 1,000 days present	Random Forests, machine learning algorithms using a two-staged approach to the ever/never outcome and days of therapy per encounter
Different: Population	Hospitals: 449 hospitals in the United States<br><br>Age: Adult and Pediatric locations<br><br>NHSN Locations included:<br>Adult Medical Ward<br>Adult Surgical Ward<br>Adult Medical/Surgical Ward<br>Adult Medical Critical Care<br>Adult Surgical Critical Care<br>Adult Medical/Surgical Critical Care<br>Adult Hematology/Oncology Ward<br>Adult Step-down<br>Pediatric Medical Ward<br>Pediatric Medical/Surgical Ward<br>Pediatric Surgical Ward<br>Pediatric Medical Critical Care<br>Pediatric Medical/Surgical Critical Care	Hospitals: 3 Duke Health System hospitals in North Carolina<br><br>Age: Any inpatient encounter including adult, pediatric, and neonatal<br><br>NHSN Locations included:<br>Any location mapped to an inpatient NHSN unit category<br><br>Models also assessed encounters with days present on a ward (Medical, Surgical, or Medical/Surgical ward) or ICU (Medical, Surgical, or Medical/Surgical Critical Care) (eTable 2)
Different: Variables used	Facility- or location-level variables collected from the NHSN annual survey and AU Option (N=7)	Encounter-level variables derived from the electronic health record (Table 1, N=204)
Different: Model performance measure	Not reported in 2017 analysis,[5] previously pseudo-R squared[1]	Area Under the Curve (AUC) for ever/never outcome and Absolute Error in days of therapy

© 2021 Moehring RW et al. *JAMA Network Open.*

eTable 4. Adult Models AUC Values

SAAR Group	Location	Tier	Lower 95% CI	AUC	Upper 95% CI
All antibacterials	ALL	1	0.820	0.825	0.829
All antibacterials	ALL	2	0.869	0.873	0.877
All antibacterials	ALL	3	0.870	0.874	0.878
All antibacterials	ALL	4	0.884	0.888	0.891
All antibacterials	ICU	1	0.774	0.791	0.809
All antibacterials	ICU	2	0.822	0.838	0.853
All antibacterials	ICU	3	0.819	0.834	0.850
All antibacterials	ICU	4	0.831	0.847	0.862
All antibacterials	WARD	1	0.777	0.783	0.790
All antibacterials	WARD	2	0.839	0.845	0.851
All antibacterials	WARD	3	0.836	0.842	0.848
All antibacterials	WARD	4	0.855	0.860	0.865
C. difficile agents (CDI)	ALL	1	0.787	0.792	0.798
C. difficile agents (CDI)	ALL	2	0.839	0.843	0.848
C. difficile agents (CDI)	ALL	3	0.845	0.850	0.854
C. difficile agents (CDI)	ALL	4	0.864	0.868	0.873
C. difficile agents (CDI)	ICU	1	0.698	0.718	0.738
C. difficile agents (CDI)	ICU	2	0.741	0.760	0.779
C. difficile agents (CDI)	ICU	3	0.742	0.761	0.780
C. difficile agents (CDI)	ICU	4	0.752	0.771	0.789
C. difficile agents (CDI)	WARD	1	0.741	0.749	0.757
C. difficile agents (CDI)	WARD	2	0.808	0.815	0.822
C. difficile agents (CDI)	WARD	3	0.810	0.817	0.823
C. difficile agents (CDI)	WARD	4	0.834	0.840	0.846
Community-onset	ALL	1	0.776	0.782	0.788
Community-onset	ALL	2	0.831	0.836	0.841
Community-onset	ALL	3	0.837	0.842	0.847
Community-onset	ALL	4	0.857	0.862	0.867
Community-onset	ICU	1	0.671	0.693	0.715
Community-onset	ICU	2	0.732	0.752	0.773
Community-onset	ICU	3	0.737	0.757	0.777
Community-onset	ICU	4	0.749	0.769	0.788
Community-onset	WARD	1	0.740	0.748	0.757
Community-onset	WARD	2	0.807	0.814	0.821
Community-onset	WARD	3	0.810	0.817	0.824
Community-onset	WARD	4	0.836	0.842	0.849
Anti-fungal	ALL	1	0.852	0.863	0.874
Anti-fungal	ALL	2	0.882	0.891	0.900
Anti-fungal	ALL	3	0.891	0.900	0.908
Anti-fungal	ALL	4	0.887	0.896	0.905
Anti-fungal	ICU	1	0.820	0.845	0.871

© 2021 Moehring RW et al. *JAMA Network Open.*

Anti-fungal	ICU	2	0.856	0.877	0.898
Anti-fungal	ICU	3	0.855	0.877	0.898
Anti-fungal	ICU	4	0.858	0.880	0.902
Anti-fungal	WARD	1	0.791	0.810	0.829
Anti-fungal	WARD	2	0.835	0.851	0.867
Anti-fungal	WARD	3	0.837	0.854	0.870
Anti-fungal	WARD	4	0.835	0.851	0.868
Resistant gram positive	ALL	1	0.858	0.863	0.868
Resistant gram positive	ALL	2	0.893	0.897	0.902
Resistant gram positive	ALL	3	0.893	0.897	0.901
Resistant gram positive	ALL	4	0.906	0.910	0.913
Resistant gram positive	ICU	1	0.798	0.814	0.830
Resistant gram positive	ICU	2	0.836	0.850	0.864
Resistant gram positive	ICU	3	0.830	0.845	0.860
Resistant gram positive	ICU	4	0.848	0.861	0.875
Resistant gram positive	WARD	1	0.811	0.820	0.828
Resistant gram positive	WARD	2	0.862	0.869	0.876
Resistant gram positive	WARD	3	0.861	0.867	0.874
Resistant gram positive	WARD	4	0.875	0.881	0.888
Hospital-onset	ALL	1	0.836	0.845	0.853
Hospital-onset	ALL	2	0.877	0.884	0.891
Hospital-onset	ALL	3	0.883	0.890	0.896
Hospital-onset	ALL	4	0.899	0.905	0.911
Hospital-onset	ICU	1	0.773	0.796	0.819
Hospital-onset	ICU	2	0.804	0.824	0.844
Hospital-onset	ICU	3	0.807	0.827	0.847
Hospital-onset	ICU	4	0.820	0.839	0.858
Hospital-onset	WARD	1	0.768	0.783	0.798
Hospital-onset	WARD	2	0.829	0.840	0.852
Hospital-onset	WARD	3	0.841	0.853	0.864
Hospital-onset	WARD	4	0.850	0.861	0.873
Narrow spectrum beta-lactam (Narrow BL)	ALL	1	0.852	0.857	0.862
Narrow spectrum beta-lactam (Narrow BL)	ALL	2	0.872	0.876	0.881
Narrow spectrum beta-lactam (Narrow BL)	ALL	3	0.871	0.876	0.881
Narrow spectrum beta-lactam (Narrow BL)	ALL	4	0.883	0.887	0.892
Narrow spectrum beta-lactam (Narrow BL)	ICU	1	0.795	0.817	0.839
Narrow spectrum beta-lactam (Narrow BL)	ICU	2	0.825	0.846	0.867
Narrow spectrum beta-lactam (Narrow BL)	ICU	3	0.815	0.837	0.858
Narrow spectrum beta-lactam (Narrow BL)	ICU	4	0.830	0.849	0.869
Narrow spectrum beta-lactam (Narrow BL)	WARD	1	0.825	0.834	0.842
Narrow spectrum beta-lactam (Narrow BL)	WARD	2	0.844	0.853	0.861
Narrow spectrum beta-lactam (Narrow BL)	WARD	3	0.841	0.850	0.858
Narrow spectrum beta-lactam (Narrow BL)	WARD	4	0.855	0.863	0.871

© 2021 Moehring RW et al. *JAMA Network Open.*

AUC Values with 95% Confidence Intervals

eTable 5. Pediatric Models AUC Values

SAAR Group	Location	Tier	Lower 95% CI	AUC	Upper 95% CI
All Antibacterials	ALL	1	0.885	0.893	0.902
All Antibacterials	ALL	2	0.902	0.910	0.918
All Antibacterials	ALL	3	0.906	0.914	0.922
All Antibacterials	ALL	4	0.926	0.933	0.939
All Antibacterials	ICU	1	0.761	0.796	0.832
All Antibacterials	ICU	2	0.784	0.818	0.852
All Antibacterials	ICU	3	0.808	0.840	0.872
All Antibacterials	ICU	4	0.829	0.859	0.889
All Antibacterials	WARD	1	0.721	0.750	0.779
All Antibacterials	WARD	2	0.775	0.801	0.826
All Antibacterials	WARD	3	0.789	0.814	0.839
All Antibacterials	WARD	4	0.805	0.829	0.853
Azithromycin	ALL	1	0.880	0.907	0.935
Azithromycin	ALL	2	0.902	0.927	0.951
Azithromycin	ALL	3	0.920	0.939	0.958
Azithromycin	ALL	4	0.926	0.944	0.963
C. difficile agents (CDI)	ALL	1	0.891	0.902	0.913
C. difficile agents (CDI)	ALL	2	0.924	0.932	0.940
C. difficile agents (CDI)	ALL	3	0.928	0.936	0.943
C. difficile agents (CDI)	ALL	4	0.934	0.942	0.949
C. difficile agents (CDI)	ICU	1	0.776	0.815	0.855
C. difficile agents (CDI)	ICU	2	0.787	0.825	0.863
C. difficile agents (CDI)	ICU	3	0.783	0.822	0.860
C. difficile agents (CDI)	ICU	4	0.804	0.840	0.876
C. difficile agents (CDI)	WARD	1	0.667	0.710	0.753
C. difficile agents (CDI)	WARD	2	0.774	0.810	0.846
C. difficile agents (CDI)	WARD	3	0.791	0.823	0.855
C. difficile agents (CDI)	WARD	4	0.776	0.812	0.848
Broad Community-onset (Broad CO)	ALL	1	0.869	0.883	0.897
Broad Community-onset (Broad CO)	ALL	2	0.911	0.921	0.932
Broad Community-onset (Broad CO)	ALL	3	0.913	0.923	0.933
Broad Community-onset (Broad CO)	ALL	4	0.922	0.932	0.941
Broad Community-onset (Broad CO)	ICU	1	0.719	0.771	0.822
Broad Community-onset (Broad CO)	ICU	2	0.715	0.768	0.822
Broad Community-onset (Broad CO)	ICU	3	0.751	0.798	0.845
Broad Community-onset (Broad CO)	ICU	4	0.754	0.801	0.849
Broad Community-onset (Broad CO)	WARD	1	0.680	0.730	0.779
Broad Community-onset (Broad CO)	WARD	2	0.794	0.836	0.879
Broad Community-onset (Broad CO)	WARD	3	0.804	0.842	0.879
Broad Community-onset (Broad CO)	WARD	4	0.821	0.857	0.893
Anti-fungal	ALL	1	0.909	0.933	0.957

© 2021 Moehring RW et al. *JAMA Network Open.*

Anti-fungal	ALL	2	0.946	0.959	0.972
Anti-fungal	ALL	3	0.959	0.970	0.981
Anti-fungal	ALL	4	0.965	0.974	0.982
Gram positive	ALL	1	0.887	0.901	0.915
Gram positive	ALL	2	0.913	0.925	0.937
Gram positive	ALL	3	0.921	0.932	0.943
Gram positive	ALL	4	0.938	0.946	0.955
Gram positive	ICU	1	0.766	0.810	0.855
Gram positive	ICU	2	0.807	0.844	0.880
Gram positive	ICU	3	0.821	0.855	0.889
Gram positive	ICU	4	0.835	0.870	0.904
Gram positive	WARD	1	0.715	0.768	0.820
Gram positive	WARD	2	0.765	0.812	0.858
Gram positive	WARD	3	0.772	0.817	0.863
Gram positive	WARD	4	0.811	0.851	0.890
Hospital-onset	ALL	1	0.924	0.938	0.951
Hospital-onset	ALL	2	0.953	0.962	0.971
Hospital-onset	ALL	3	0.956	0.964	0.972
Hospital-onset	ALL	4	0.952	0.961	0.971
Hospital-onset	ICU	1	0.862	0.900	0.938
Hospital-onset	ICU	2	0.886	0.915	0.945
Hospital-onset	ICU	3	0.871	0.906	0.940
Hospital-onset	ICU	4	0.903	0.928	0.953
Narrow Community-onset (Narrow CO)	ALL	1	0.869	0.882	0.895
Narrow Community-onset (Narrow CO)	ALL	2	0.884	0.896	0.908
Narrow Community-onset (Narrow CO)	ALL	3	0.881	0.894	0.906
Narrow Community-onset (Narrow CO)	ALL	4	0.923	0.932	0.941
Narrow Community-onset (Narrow CO)	ICU	1	0.619	0.683	0.748
Narrow Community-onset (Narrow CO)	ICU	2	0.627	0.692	0.757
Narrow Community-onset (Narrow CO)	ICU	3	0.725	0.782	0.840
Narrow Community-onset (Narrow CO)	ICU	4	0.773	0.826	0.879
Narrow Community-onset (Narrow CO)	WARD	1	0.652	0.700	0.748
Narrow Community-onset (Narrow CO)	WARD	2	0.703	0.745	0.788
Narrow Community-onset (Narrow CO)	WARD	3	0.709	0.754	0.800
Narrow Community-onset (Narrow CO)	WARD	4	0.772	0.810	0.849

AUC Values with 95% Confidence Intervals

© 2021 Moehring RW et al. *JAMA Network Open.*

eReferences

1. van Santen KL, Edwards JR, Webb AK, et al. The Standardized Antimicrobial Administration Ratio: A New Metric for Measuring and Comparing Antibiotic Use. *Clinical infectious diseases : an official publication of the Infectious Diseases Society of America.* 2018;67(2):179-185.
2. Moehring RW, Dodds Ashley ES. Developing Patient Safety Outcome Measures and Measurement Tools for Antibiotic Stewardship Programs: Metrics Guide. https://dason.medicine.duke.edu/system/files/page-attachments/704/dason-cdcfinalanalysistool-r11-21.pdf. Published 2017. Accessed2019.
3. Tan C, Vermeulen M, Wang X, Zvonar R, Garber G, Daneman N. Variability in antibiotic use across Ontario acute care hospitals. *The Journal of antimicrobial chemotherapy.* 2017;72(2):554-563.
4. Agency for Healthcare Research and Quality: Beta Clinical Classifications Softward (CCS) for ICD-10-CM/PCS. https://www.hcup-us.ahrq.gov/toolssoftware/ccs10/ccs10.jsp. Published 2018. Accessed2019.
5. O'Leary EN, Edwards JR, Srinivasan A, et al. National Healthcare Safety Network Standardized Antimicrobial Administration Ratios (SAARs): A Progress Report and Risk Modeling Update Using 2017 Data. *Clinical Infectious Diseases.* 2020.

© 2021 Moehring RW et al. *JAMA Network Open.*
